# Vasorelaxant Effects Induced by Red Wine and Pomace Extracts of Magliocco Dolce *cv*.

**DOI:** 10.3390/ph13050087

**Published:** 2020-05-03

**Authors:** Gabriele Carullo, Amer Ahmed, Fabio Fusi, Fabio Sciubba, Maria Enrica Di Cocco, Donatella Restuccia, Umile Gianfranco Spizzirri, Simona Saponara, Francesca Aiello

**Affiliations:** 1 Department of Pharmacy, Health and Nutritional Sciences, Department of Excellence 2018-2022, University of Calabria, Ed. Polifunzionale, 87036 Rende (CS), Italy; gabriele.carullo@unical.it (G.C.); donatella.restuccia@unical.it (D.R.); g.spizzirri@unical.it (U.G.S.); 2 Department of Life Sciences, University of Siena, Via Aldo Moro 2, 53100 Siena, Italy; aa.biotechiub@gmail.com; 3 Department of Biotechnology, Chemistry and Pharmacy, Department of Excellence 2018-2022, University of Siena, Via Aldo Moro 2, 53100 Siena, Italy; fabio.fusi@unisi.it; 4 Department of Chemistry, University of Rome La Sapienza, Piazzale Aldo Moro 5, 00185 Roma, Italy; fabio.sciubba@uniroma1.it (F.S.); mariaenrica.dicocco@uniroma1.it (M.E.D.C.)

**Keywords:** vasoactivity, grape polyphenols, flavonoids, nitric oxide synthase, endothelium, TRPV1

## Abstract

Several epidemiological studies demonstrate that moderate (red) wine consumption may afford protection against cardiovascular diseases. Protection is ascribed to the biological activity of wine components, many of which, however, are discarded during winemaking. In vitro rat thoracic aorta rings contracted with phenylephrine or KCl were used to assess the vasorelaxant activity of extracts from wine pomaces (seeds and skins) of the Calabrian autochthonous grape variety Magliocco dolce (Arvino). NMR spectroscopy was used to ascertain their chemical composition. Data demonstrate that seed and skin, but not must, extracts are capable of relaxing vascular preparations in an endothelium-dependent manner, similarly to the red wine extract, due to the presence of comparable amounts of bioactive constituents. In rings pre-contracted with 20–30 mM KCl, only seed extracts showed a moderate relaxation. The most efficacious vasodilating extract (wine) showed a good antioxidant profile in both [(2,2-diphenyl-1-picrylhydrazyl)acid] radical (DPPH) and [2,2’-azino-bis (3-ethylbenzothiazoline-6-sulphonic acid)] radical (ABTS) assays. In conclusion, winemaking from Magliocco dolce grape can provide potentially health-promoting by-products useful in cardiovascular disease management.

## 1. Introduction

Several plant extracts and functional foods have been developed over the years to treat various ailments, including cardiovascular diseases [1]. Cardiovascular diseases represent the first cause of death worldwide [2]. In this scenario, hypertension plays a key role as the major risk factor associated with coronary, ischemic, rheumatic heart diseases, and other cardiovascular complications. Unfortunately, first choice anti-hypertensive drugs are associated with several unwanted side effects that hamper patient adherence to therapy [3]. Over the years, foods and food-derived products have emerged as new tools in hypertension management, particularly at the pre-hypertension stage because of their nutraceutical properties [4]. Mediterranean food and beverages, including grain, vegetables, milk, cheese, chicken, egg, fish, soybean, tea, wine, grapes, and mushrooms represent promising choices [5,6]. In fact, they contain various amount of vitamin C and E, angiotensin II-converting enzyme (ACE) inhibitory peptides, flavonoids, flavanols, anthocyanins, phenolic acids, tannins, and resveratrol, which may reduce high blood pressure through different mechanisms, including ACE inhibition, antioxidant, vasodilatory, and Ca^2+^ channel blocking activities, as well as the stimulation of endothelial nitric oxide (NO) release [7]. Nowadays, several scientific evidences and epidemiological studies support the hypothesis that moderate wine consumption (especially red wine) is a healthy “cardio-protective” strategy [8]. Unlike white wine, red wine is prepared by the alcoholic fermentation of must in the presence of skin and seeds that rise its content of phenolic compounds. Flavonoids, including monomeric (flavones, flavonols, anthocyanins, flavan-3-ols, catechins, and epicatechin), dimeric (salicin), or polymeric compounds (tannins), account for over 85% of the phenolic components in red wine. Catechins and epicatechins are particularly abundant in both grape skins and seeds, accounting for up to 60% of the total phenolic content of the seeds [9,10]. Hence, grape pomaces, generally discarded as by-products of the winemaking process, deserve a careful evaluation [11]. In fact, polyphenols promote cellular and molecular mechanisms that drive anti-inflammatory, antioxidant, and hypotensive responses [12]. In this context, various flavonoids exhibit direct vasodilating activities in isolated arteries. Quercetin and kaempferol, for example, show a not yet fully understood, endothelium-dependent vasodilation that does not characterize structurally similar compounds [13,14]. In this field, it is worth underlining that not only single wine components, but also grape, pomace, and wine extracts can induce endothelium-dependent vasorelaxation by stimulating endothelial nitric oxidase synthase (eNOS) and/or prolonging the biological activity of NO [9]. These effects are often caused by the synergic interaction of mixture components rather than by a single chemical entity. Therefore, a rationale pomace management could lead to a valuable dietary approach to fight cardiovascular diseases. Investigation of scarcely exploited autochthonous cultivars might represent a good opportunity to find novel functional food material. The native Calabrian cultivar, Magliocco dolce wine variety, recently registered (7 June 2019) in the National Wine Variety Register by decree of Italian Ministry of Agricultural Policies [15], was therefore selected for this purpose. Berry skin and seeds extracts, must, and wine were evaluated for their in vitro vasorelaxant activity. Findings presented here suggest that Magliocco dolce harbors a unique chemical composition endowed with potential vasoactivity. Industry could take advantage of these ingredients [16,17] to develop novel functional food additives.

## 2. Results

### 2.1. Extraction and ^1^H NMR Characterization of the Extracts

**Figure 1:**
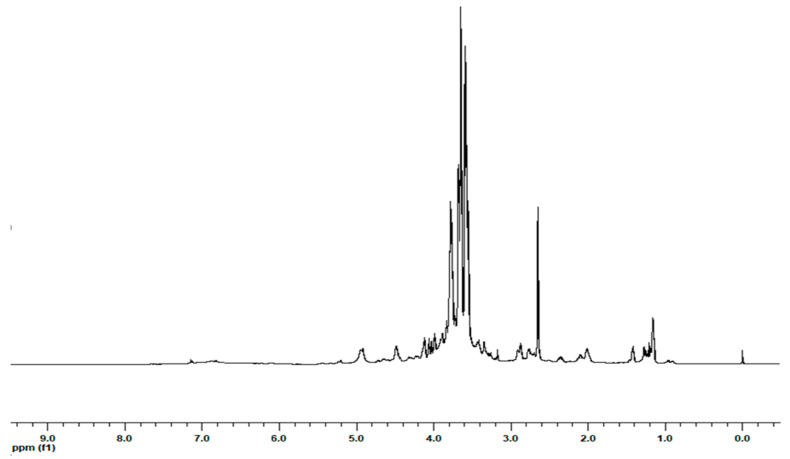
Representative 1D NMR spectrum of LDC3.

The extracts (**LDC1**, **2**, **5**, and **6**) were prepared from Magliocco dolce wine variety at rt, with a procedure previously used to extract biomolecules from red wine pomace. The obtained extracts were named according to our house style, namely **LDC1** (skin extract after rosé vinification process), **LDC2** (skin extract after red vinification process), **LDC5** (seed extract after rosé vinification process), **LDC6** (seed extract after red vinification process). Red wine and must were filtered, evaporated, and freeze-dried obtaining **LDC3** (freeze-dried red wine, 1D NMR Figure 1) and **LDC4** (freeze-dried red must). The different **LDC** samples obtained from Magliocco dolce wine variety were analyzed by ^1^H NMR for their chemical compositions searching for chemical entities likely responsible for vasoactivity (Table 1). In the SI, the NMR peaks are identified (Table S1) and original NMR spectra for all the samples are reported (Figures S1–S8).

Apparently, **LDC** extracts contained variable quantities of amino acids, organic acids, carbohydrates, and flavonoids. In particular, **LDC1** contained amino acids such as alanine and GABA, organic acids (i.e., quinic and malic acids), glucose, and traces of glycosylated flavonoids. **LDC2** contained a wide variety of amino and organic acids with traces of catechins and flavonoids. Of interest, **LDC3** included a relatively high concentration of GABA, gallic acid, and flavonoids. **LDC4** appeared rich in amino acids and glucose, but devoid of catechin and glycosylated flavonoids. Similar to **LDC3**, **LDC5** and **LDC6** seed extracts showed a high content of polyphenols, such as catechin and gallic acid.

### 2.2. Effect of LDC Extracts on Phenylephrine-Induced Contraction

**Figure 2:**
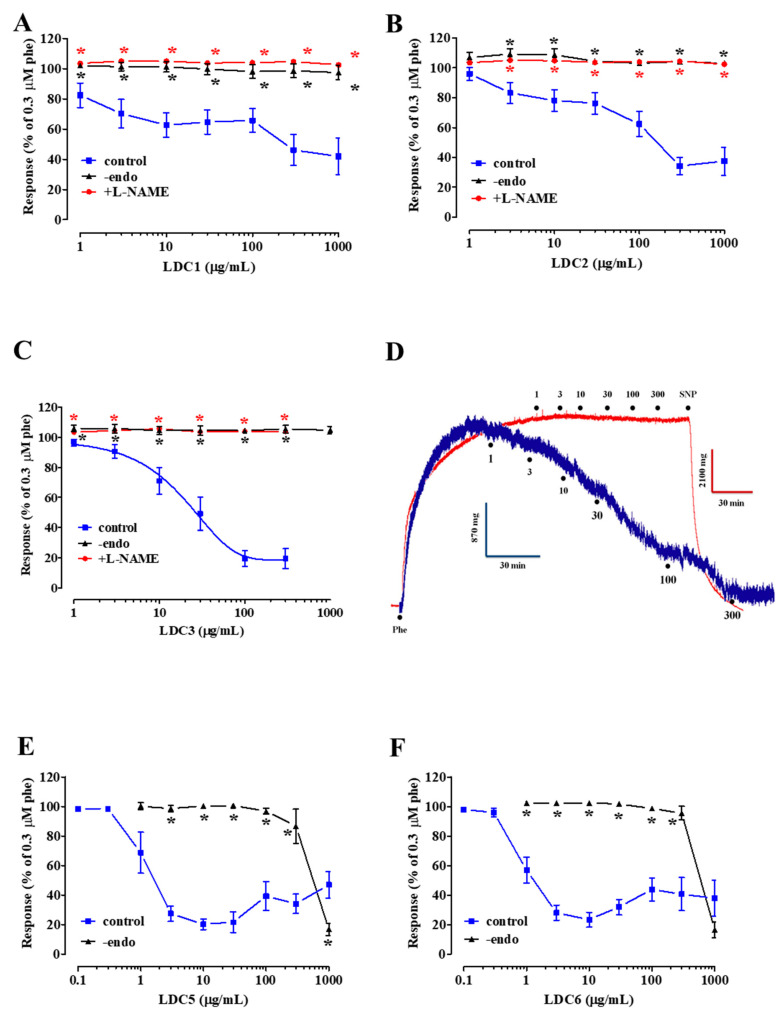
Effects of **LDC** extracts on phenylephrine-induced contraction in either endothelium-intact or deprived rat aorta rings. Endothelium-intact (control) or -denuded (-endo) rings, pre-contracted by 0.3 µM phenylephrine, were challenged with cumulative concentrations of (**A**) **LDC1**, (**B**) **LDC2**, (**C**) **LDC3**, (**E**) **LDC5**, and (**F**) **LDC6**. Some experiments were performed in endothelium-intact rings pre-incubated with 100 µM L-NAME. (**D**) Traces representative of 5–6 similar experiments, showing the effects of **LDC3** on endothelium-intact rings in the absence (blue trace) or presence of 100 µM L-NAME (red trace). In the ordinate scale, relaxation is reported as a percentage of the initial tension induced by phenylephrine (Phe). Data points represent mean ± SEM (*n* = 4–8). *P < 0.05 vs. control, one-way ANOVA or Student’s t test for unpaired samples.

In rings with an intact endothelium, all **LDC** extracts except **LDC4** caused a concentration-dependent relaxation of phenylephrine-induced contraction. E_max_ values were 58.1 ± 12.1% (1 mg/mL **LDC1**, *n* = 5), 65.8 ± 5.8% (0.3 mg/mL **LDC2**, *n* = 7), 80.6 ± 5.3% (0.1 mg/mL **LDC3**, *n* = 6), 79.8 ± 3.6% (10 µg/mL **LDC5**, *n* = 6), and 76.6 ± 5.1% (10 µg/mL **LDC6**, *n* = 7) (Figure 2A–F). Skin extracts **LDC1** and **LDC2** showed a biphasic relaxing behavior, with a primary activity plateauing, and a secondary effect starting at concentrations of 300 µg/mL and 100 µg/mL, respectively (Figure 2A,B). Seed extracts **LDC5** and **LDC6** were the most potent of the series (IC_50_ values of 1.7 ± 0.3 μg/mL, *n* = 6, and 1.3 ± 0.3 μg/mL, *n* = 7, respectively; Figure 2) but, at concentrations >10 µg/mL, they showed a hormetic behavior giving rise to vessel contraction. Finally, even the lyophilized red wine **LDC3** markedly relaxed aortic rings with an IC_50_ value of 39.1 ± 13.9 μg/mL (*n* = 6).

**Figure 3:**
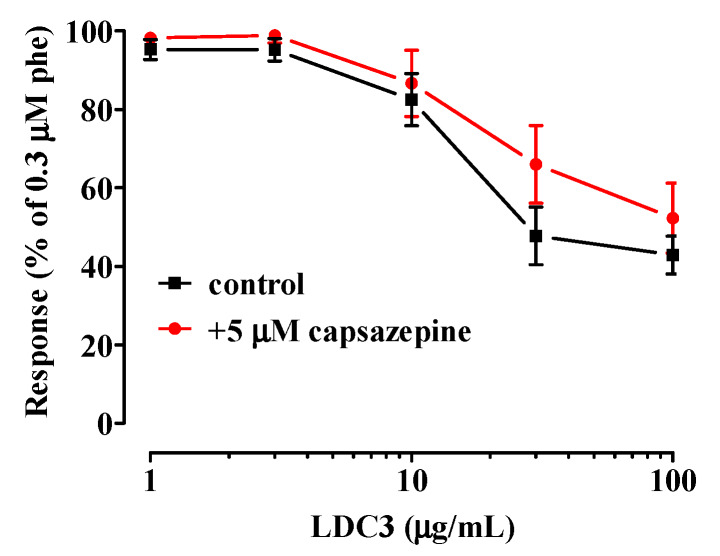
Effects of capsazepine on **LDC3**-induced relaxation. Endothelium-intact rings, pre-contracted by 0.3 µM phenylephrine, were challenged with cumulative concentrations of **LDC3**, in the absence (control) or presence of 5 µM capsazepine. In the ordinate scale, relaxation is reported as a percentage of the initial tension induced by phenylephrine (Phe). Data points represent mean ± SEM (*n* = 6).

In endothelium-deprived preparations, **LDC** extracts did not cause vasorelaxation; only **LDC5** and **LDC6**, at the maximal concentration assessed (1 mg/mL), significantly reverted phenylephrine-induced active tone (E_max_ of 83.1 ± 4.1% and 83.5 ± 5.3%, respectively, *n* = 5; Figure 2E,F). The three most interesting extracts of the series (i.e., effective and devoid of a hormetic behaviour), namely **LDC1-LDC3**, were further investigated in order to shed light on the mechanism underpinning their endothelium-dependent antispasmodic activity. Pre-exposure of endothelium-intact rings to L-NAME antagonised the vasorelaxant activity of **LDC1**, **LDC2**, and **LDC3** (Figure 2A–D). Finally, vasorelaxation induced by the most effective mixture, i.e., **LDC3**, was assessed in the presence of a TRPV1 channel blocker: capsazepine did not affect **LDC3**-induced spasmolysis in endothelium-intact preparations (Figure 3).

### 2.3. Effects of LDC Extracts on High KCl-Induced Contraction

**Figure 4:**
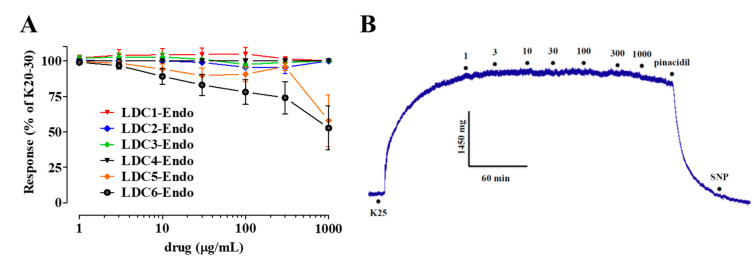
Effect of **LDC** extracts on high KCl-induced contraction of rat aorta rings. (**A**) Concentration-response curves for **LDC** extracts were constructed on endothelium-denuded preparations pre-contracted by 20–30 mM KCl. In the ordinate scale, relaxation is reported as percentage of the initial tension induced by KCl, taken as 100%. In the abscissa, the concentration of each drug (as specified in the legend to symbols) is reported in µg/mL. Data points represent mean ± SEM (*n* = 4–6). (**B**) Trace representative of 4 similar experiments, showing the effects of **LDC1** (µg/mL) on an endothelium-denuded ring pre-contracted by 25 mM KCl (K25). The effect of 100 µM pinacidil and 100 µM sodium nitroprussiate (SNP) is also shown.

The cumulative addition of the seed extracts **LDC5** and **LDC6** to endothelium-denuded rings depolarized with 20–30 mM KCl caused a modest decrease of the active tension (E_max_ values of 42.1 ± 18.2% and 47.3 ± 15.5%, respectively; *n* = 6; Figure 4A). The remaining extracts did not affect this contraction up to the maximal concentration assessed in the present work (Figure 4A). Contrary to **LDC** extracts showing no relaxing activity, the addition of 100 µM pinacidil completely reverted vessel tone to basal level (Figure 4B).

### 2.4. Antioxidant Activity

ROS scavenging activity, metal ions chelation, and oxidant enzyme inhibition support several biological effects of phenolic compounds including protection of cellular structures and biomolecules. Disposable phenolic equivalents (TPE) of the lyophilized red wine **LDC3**, the most promising mixture in the series, expressed as milliequivalents of gallic acid per grams of **LDC3**, were evaluated by the Folin–Ciocalteu procedure, detecting the transfer of electrons from phenolic compounds to the reagent in an alkaline environment.

**Figure 5:**
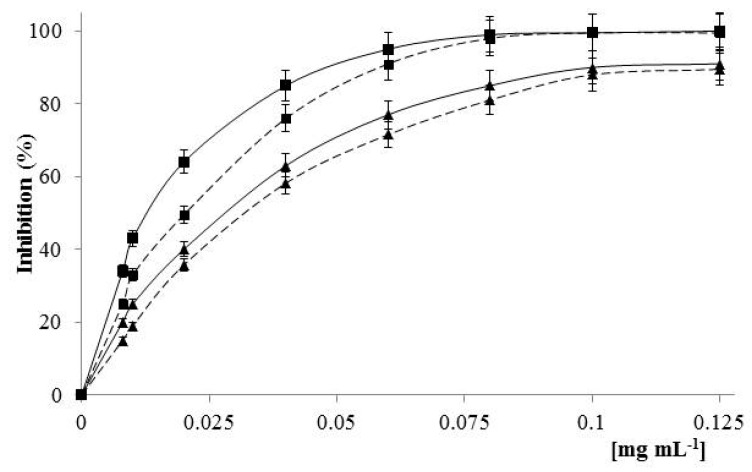
Scavenging activity of **LDC3** (- - - -) against ABTS (∎) and DPPH (▲) radicals. Ascorbic acid (w/v) (^_____^) was used as positive control.

The TPE assay provides a good estimate of the phenolic content, though some non-phenolic chemical structures having reducing capacity, such as organic sugars and acids, may overestimate the amount of total phenolic detected. The TPE value recorded was 0.306 meq gallic acid per gram of **LDC3**. Data related to the antioxidant capacity of **LDC3** were expressed as total antioxidant capacity (TAC) and scavenging activity in aqueous and organic media. Specifically, TAC value was 0.512 meq catechin per gram of **LDC3**. This result was in agreement with the TPE value, indicating that TAC can be essentially ascribed to the phenolic molecules. Figure 4 and Figure 5 depicts the scavenging activity of **LDC3** against DPPH and ABTS radical species. These data suggest that **LDC3** antioxidant activity was more prominent in aqueous rather than organic medium (IC_50_ 1.5-fold higher). In particular, the calculated IC_50_ values were 0.020 mg mL^−1^ and 0.033 mg mL^−1^ against ABTS and DPPH radicals, respectively.

## 3. Discussion

The main findings of the present work can be summarized as follows:

1. Magliocco dolce wine and pomaces (obtained from different steps during the winemaking process) comprise a range of valuable bioactive entities (e.g., polyphenols);
2. **LDC** extracts, except **LDC4**, contain vasorelaxant agents capable of relaxing in vitro vascular preparations in an endothelium-dependent manner;
3. relaxation induced by **LDC1**-**LDC3** is dependent on the activation of eNOS;
4. TRPV1 channels are not involved in the relaxation caused by **LDC3**.

The endothelium is a fundamental regulator of vascular tone and blood homeostasis. In fact, endothelial cells produce a paradigm of vasoactive substances such as NO, prostacyclin, hydrogen sulfide, and endothelium-derived hyperpolarizing factor(s), which finely balance vascular smooth muscle tone [18]. In particular, NO synthesized by eNOS [19] triggers a plethora of molecular events in the underlining smooth muscle cells via the activation of soluble guanylyl cyclase, that eventually result in spasmolysis. Vasorelaxation elicited by **LDC1-LDC3** and **LDC5-LDC6** was clearly endothelium-dependent, as it was not observed in endothelium-denuded preparations. In addition, spasmolysis operated by **LDC1-LDC3** involved the activation of eNOS, because pre-exposure of the rings to L-NAME (an eNOS inhibitor) completely abolished this phenomenon. Though the functional evidences here presented strongly sustain this hypothesis, only Western blot analysis of eNOS expression levels and phosphorylation pattern will directly substantiate enzyme activation by these extracts. eNOS is constitutively expressed in the endothelial cells and tightly regulated at transcriptional and posttranslational level. For instance, extracellular Ca^2+^ entering through the TRPV1 channels, binds to calmodulin: the Ca^2+^-calmodulin complex, in turn, releases caveolin-1 from eNOS, thus enhancing its activity [18]. The TRPV1 channel, however, was not involved in the activation of eNOS operated by **LDC3**, as capsazepine, a specific blocker of this channel, did not affect **LDC3**-induced spasmolysis. This implies the involvement of other mechanisms, e.g., phosphorylation at Ser^1177^ by several kinases such as Akt, PKA, and AMPK among others [20]. Natural compounds including gallic acid, succinic acid, and flavonoids (such as quercetin) are capable to activate eNOS through phosphorylation of Ser^1177^. Interestingly, these molecules were found in the **LDC3** sample and hence might concur to its documented eNOS-dependent vasorelaxation. Further support to this hypothesis arises from the observation that **LDC4**, lacking many of these constituents, was devoid of relaxing activity.

Opening of K^+^ channels, and the consequent flux of K^+^ towards the extracellular milieu, hyperpolarizes the vascular smooth muscle cell membrane, thus closing voltage-dependent Ca^2+^ channels and causing vasorelaxation. K^+^ channel openers antagonize the contraction induced, in vitro, by a moderate increase (from 5 mM to approximately 20–30 mM) of the extracellular KCl concentration. Therefore, the observation that **LDC1-LDC4** failed to relax rings pre-contracted with 20–30 mM KCl indicates that these extracts may not harbor any agent capable of opening K^+^ channels, or may harbor it but not in adequate concentration. This conclusion is further supported by the fact that pinacidil, an ATP-dependent K^+^ channel opener, unlike **LDC1-LDC4** extracts, was able to revert KCl-induced active tone under the same experimental conditions. Only **LDC5** and **LDC6** showed a moderate relaxation, though only at high concentrations. This activity might be ascribed to the presence of high levels of catechin and gallic acids, which activate various K^+^ channels [21] or, more likely, to a synergistic, unspecific action of the extract components on more than one target located on the smooth muscle cell, as it was observed, though to a greater extent, also in endothelium-denuded, phenylephrine-stimulated preparations.

The alteration of biological macromolecules, such as proteins, lipids and nucleic acids is one direct consequence of oxidative stress and is associated to the onset of several chronic diseases [22,23]. In the vascular system, physiological levels of ROS are necessary to sustain endothelial homeostasis and smooth muscle cell contraction. However, uncontrolled ROS production induces vascular cell impairment, recruitment of inflammatory cells, lipid peroxidation, activation of metalloproteinases and deposition of extracellular matrix, cooperatively leading to vascular remodeling and dysfunction [24]. Therefore, it is conceivable that agents capable of scavenging ROS, thus limiting the damage caused by oxidative stress [25,26], are intensively pursued. Phenolic compounds, including flavonoids, abundantly present in grape and its derivatives, are effective ROS scavengers, inhibit pro-oxidant enzymes, and chelate metal ions (Fe^3+^ and Cu^+^) that trigger lipid peroxidation, thus representing favorable, naturally occurring antioxidants that might play an important role in promoting cardiovascular health, particularly in endothelial dysfunction-related maladies [27]. In this context, the antioxidant features of **LDC3** appear interesting, highlighting an efficacy in the aqueous medium 1.5-fold higher than that recorded in the organic medium. This finding is particularly important because the ABTS radical scavenging assay is the most reliable test to evaluate the antioxidant activity of food matrices, being able to detect accurately the response produced by hydrophilic compounds [28]. Polyphenols likely play a major role among the bioactive constituents responsible for the antioxidant features of **LDC3**. Their amount is in the same order of magnitude of that found in other Calabrian autochthonous grape derivatives [29]. Gallic acid, catechin, and glycosylated flavonoids detected in high concentrations may significantly contribute to the in vitro antioxidant capacity of the extract. In fact, they possess numerous acidic, phenolic hydroxyl groups and can establish resonance between the free electron pair on the phenolic oxygen and the benzene ring [30]. As oxidative stress is a major factor leading to endothelial dysfunction with significant prognostic implications for cardiovascular events, antioxidants, such as the polyphenols abundantly present in **LDC** extracts, can play a valuable vascular-protective role and improve endothelial function [31].

## 4. Materials and Methods

### 4.1. Chemicals

All reagents used in this study were purchased from Sigma-Aldrich Chemical Co. Ltd. (Milan, Italy) and VWR International (Milan, Italy) and, unless specified otherwise, were of analytical grade or higher. Solvents of pharmaceutical grade, including ethanol 96%, were purchased from Merck (Darmstad, Germany). Deuterated solvents and standards for NMR analysis were purchased from Sigma-Aldrich (St. Louis, MO, USA). Grape pomaces, wine, and must were supplied by Dr. Paolo Chirillo, Le Moire srl, Ctr. Strivillati, 88040 Motta Santa Lucia (Catanzaro, Italy, info@lemoire.it). Phenylephrine, acetylcholine, sodium nitroprusside, pinacidil, Nω-nitro-L-arginine methyl ester (L-NAME), capsazepine, and nifedipine were purchased from Sigma Chemical Co. (Milan, Italy). Phenylephrine was solubilized in 0.1 M HCl; L-NAME, acetylcholine, and sodium nitroprusside in distilled water, nifedipine in ethanol, and capsazepine and pinacidil in DMSO. Extracts were resuspended in a modified Krebs–Henseleit solution (KHS; see below for composition).

### 4.2. Preparation of the Arvino Grape Pomace Extracts

The by-products of the winemaking process were obtained after the red grapes had been pressed. Samples were milled and stored in polyethylene film bags packed under vacuum and stored at −20 °C until use. Briefly, the first step of the red vinification process involved removal of the stalks (i.e., the parting of the woody stalk) from the must, thus avoiding the transfer of bitter tasting tannin to wine. Then the must was transferred into the steel fermentation tanks, closed and stirred. The peels were left macerating in the must for a period of 20–30 days. Must derived from red vinification after 10 days. Seeds and skins were manually separated until extraction. The extracts were prepared using a mixture of 40% ethanol and 60% distilled water. Extraction was done with a solute/solvent ratio of 1:50 (w/v). The suspensions were shaken in a rotary shaker for 24 h at 100 rpm and at a temperature of 25 °C. Then the mixture was centrifuged at 3500xg for 25 min. Ethanol was eliminated with a rotary evaporator and the aqueous solution was freeze-dried and stored at −20 °C until use. Extractions were carried out in triplicate. Wine and must for the production of red wine were evaporated to eliminate ethanol, centrifuged, filtered, and freeze-dried. The samples were named as follows: **LDC1** (skin extract after rosé vinification process), **LDC2** (skin extract after red vinification process), **LDC3** (freeze-dried red wine), **LDC4** (freeze-dried red must), **LDC5** (seed extract after rosé vinification process), and **LDC6** (seed extract after red vinification process) [32].

### 4.3. NMR Analysis

Aliquots of **LDC** samples were analyzed by NMR spectroscopy to assess their chemical composition. In particular, the assignment of the resonances was performed by analyzing ^1^H characteristics and cross-correlated signals in 2D ^1^H-^1^H TOCSY spectra (see Supplementary Material) and by comparison with literature data [33]. Quantification of the identified compounds was performed comparing the signal integral to the reference one, and quantities were expressed in mg of compound normalized to the aliquot weight expressed in g. Each dry aliquot was dissolved in 0.6 mL of D_2_O:CD_3_OD (2:1 ratio) containing 2 mM 3-(trimethylsilyl)-propionic-2,2,3,3-d_4_ acid sodium salt as chemical shift and concentration reference. All spectra were recorded at 298 K on a Bruker AVANCE III spectrometer operating at the proton frequency of 400.13 MHz and equipped with a Bruker multinuclear z-gradient inverse probehead. ^1^H spectra were acquired using the presat pulse sequence for solvent suppression with 128 transients, a spectral width of 6000 Hz and 64K data points for an acquisition time of 5.5 s. The recycle delay was set to 9.5 s in order to achieve complete resonance relaxation between successive scansions. ^1^H-^1^H TOCSY experiments were acquired with spectral width of 6000 Hz in both dimensions, a data matrix of 8K × 256 points, mixing time of 110 ms and relaxation delay of 2s.

### 4.4. Vasoactivity Assessments of LDC Extracts

#### 4.4.1. Animals

All animal care and experimental protocols performed in the present study were in strict compliance with the European Union Guidelines for the Care and the Use of Laboratory Animals (European Union Directive 2010/63/EU) and were also approved by the Animal Care and Ethics Committee of the University of Siena and Italian Department of Health (666/2015-PR).

#### 4.4.2. Preparation of Rat Aortic Rings

Male Wistar rats, weighing 250–400 g, obtained from Charles River Italia (Calco, Italy), were anaesthetized with a gas mixture of 4% isoflurane and 96% O_2_ by using Fluovac (Harvard Apparatus, Holliston, MA, USA), decapitated and exsanguinated. Thoracic aorta was immediately removed and gently cleaned of adipose and connective tissues. Aorta rings (2.5–3.0 mm width), either endothelium-intact or -deprived (the endothelium being removed by gently rubbing the lumen of the ring with the curved tips of a forceps), were mounted horizontally between two parallel L-shaped stainless steel hooks, one of which connected to an isometric transducer. Rings were allowed to equilibrate for 60 min in KHS (composition in mM: NaCl 118; KCl 4.75; KH_2_PO_4_ 1.19; MgSO_4_ · 7 H_2_O 1.19; NaHCO_3_ 25; glucose 11.5; CaCl_2_ · 2 H_2_O 2.5; gassed with a 95% O_2_/5% CO_2_ gas mixture to create a pH of 7.4) under a passive tension of 1 g. Isometric tension was recorded using a digital PowerLab data acquisition system (PowerLab 8/30; ADInstruments) and analyzed by using LabChart 7.3.7 Pro (PowerLab; ADInstruments). Functional integrity of smooth muscle was assessed by recording the response of the rings to 0.3 µM phenylephrine. The presence of a functional or dysfunctional endothelium was indicated by a ≥75% or ≤10% acetylcholine-induced relaxation, respectively, of the phenylephrine-induced tone [34,35].

#### 4.4.3. Effect of **LDC** Extracts on Phenylephrine- and High K^+^-Induced Contraction

Rings were pre-contracted by either 0.3 µM phenylephrine or 20–30 mM KCl. Once the plateau was achieved, **LDC** extracts were added cumulatively. In some experiments, preparations were pre-incubated with either 100 µM L-NAME (eNOS inhibitor) for 30 min, or with 5 µM capsazepine (selective TRPV1 channel blocker) for 20 min [33]. Then, phenylephrine was added to the organ bath to elicit contraction and the effects of **LDC** extracts assessed always in the presence of the inhibitor or blocker. At the end of the experimental protocols, the NO-donor sodium nitroprusside (100 µM) alone (phenylephrine-pre-contracted rings) or the Ca_V_1.2 channel blocker nifedipine (1 µM) followed by sodium nitroprusside (KCl-pre-contracted rings) were added to test the functional integrity of smooth muscle. **LDC**-induced relaxation was calculated as a percentage of the contraction evoked by either 0.3 µM phenylephrine or 20–30 mM KCl (taken as 100%) [36].

### 4.5. Antioxidant Activity of LDC3

Antioxidant activity of **LDC3** was evaluated as total phenolic equivalents (TPE), total antioxidant capacity (TAC) and scavenging activity against DPPH and ABTS radical species.

#### 4.5.1. Total Phenolic Equivalents (TPE) by Folin–Ciocalteu Procedure

Folin–Ciocalteu procedure was used to determine total phenolic equivalents (TPE), expressed as milliequivalents of gallic acid per grams of **LDC3** [37]. Briefly, 6.0 mL of a hydro alcoholic solution (50/50 v/v) of **LDC3** were placed in a volumetric flask (10 mL) and then Folin–Ciocalteu reagent (1 mL) was added. After 3 min, 3.0 mL of Na_2_CO_3_ were added, and the mixture allowed to stand for 2 h under intermittent shaking. The absorbance was measured at 760 nm against a control sample made of the sole hydro alcoholic mixture (50/50 v/v), under the same reaction conditions. A calibration curve was built using five different standard solutions (8, 16, 24, 32, and 40 μM gallic acid) and the correlation coefficient (R^2^), slope, and intercept of the linear regression equation obtained calculated by the method of least square. Each measurement was performed in triplicate and data expressed as means (±SD). UV-Vis absorption spectra were recorded with a Jasco V-530 UV/Vis spectrometer (Jasco, Tokyo, Japan).

#### 4.5.2. Assessment of Total Antioxidant Capacity (TAC)

The total antioxidant capacity of **LDC3** was determinted as described by Cirillo et al. [38] with slight modifications and expressed as catechin equivalent concentration. Briefly, 0.3 mL of hydro alcoholic solution (50/50 v/v) of **LDC3** were added to 1.2 mL of reagent solution (0.6 M H_2_SO_4_, 28.0 M Na_3_PO_4_, and 4.0 M (NH_4_)_2_MoO_4_). The reaction mixture was incubated at 95 °C for 150 min and after cooling to room temperature, the absorbance measured at 695 nm against a control, under the same reaction conditions. By using five different catechin standard solutions (8, 16, 24, 32, and 40 μM), a calibration curve was built and the correlation coefficient (R^2^), slope, and intercept of the linear regression equation obtained by the method of least-squares calculated. Each measurement was performed in triplicate and data expressed as means (±SD).

#### 4.5.3. Assessment of Scavenging Activity against DPPH Radicals

Free radical scavenging properties of **LDC3** were estimated towards DPPH [39]. Briefly, 1.0 mL of hydro alcoholic solution (50/50 v/v) of **LDC3** was added to a 10 mL volumetric flask, along with 4.0 mL of hydro alcoholic solution (50/50 v/v) and 5.0 mL of ethanol solution of DPPH (200 μM), obtaining a solution of DPPH with a final concentration of 100 μM. The solution was incubated at 25 °C and, after 24 h, the absorbance of the remaining DPPH was read at 517 nm. Ascorbic acid (0.1 g/100 mL; w/v) was used as positive control. The scavenging activity of **LDC3** was measured as the decrease in absorbance of the sample and expressed as percent blockade of DPPH radicals calculated according to the following Equation (1):

(1) $$inibition\;\%=\frac{A_{0}-A_{1}}{A_{0}}\times 100$$

where A_0_ is the absorbance of a standard without **LDC3**, while A_1_ is the absorbance of the sample containing **LDC3**. Each measurement was carried out in triplicate and data expressed as means (± SD).

#### 4.5.4. Assessment of Scavenging Activity against the ABTS Radical Cations

The free radical scavenging property of **LDC3** in aqueous media against ABTS was assessed [40]. ABTS radical cation (ABTS^+^) solution (7.0 mM) was left in the dark at room temperature for 12–16 h, and then the concentration adjusted to attain an absorbance of 0.970 ± 0.020 at 734 nm. The scavenging effect of **LDC3** was evaluated by adding 500 µL of its hydroalcoholic solution (50:50 v/v) to 2.0 mL of the ABTS^+^ solution. The mixture was incubated in a water bath at 37 °C protected from light. The decrease in absorbance at 734 nm was measured after 5 min. Ascorbic acid (0.1 g/100 mL; w/v) was used as positive control. The antioxidant activity was expressed according to equation (1). All samples were assayed in triplicate and data expressed as means (±SD).

### 4.6. Data Analysis

Graghpad Prism version 5.04 (GraphPad Software Inc., San Diego, CA, USA) was used to analyze the vasoactivity data. Data are reported as mean ± SEM; n, indicated in parentheses, is the number of rings isolated from at least three independent animals. Statistical analysis was performed by either one-way ANOVA followed by Dunnett’s post-hoc test, or by unpaired Student’s t-test (two tailed) using Graghpad Prism software: *P* < 0.05 was considered significant. Where possible, nonlinear regression analysis was performed to calculate the IC_50_ value.

## 5. Conclusions

In conclusion, the present results demonstrate the potential vasorelaxant activity of the as yet unexplored Magliocco dolce pomace, comparable to that of the freeze-dried red wine. Noticeably, this activity is dependent on the activation of eNOS and seems to be linked to the chemical composition of the extract. Further experiments are necessary to recognize whether pomace can represent a better source of vasoactive molecules than wine. Vasoactivity, along with the antioxidant property of the constituents, makes Magliocco dolce pomace an interesting matrix worth being developed into novel functional food additives to improve human health associated to the cardiovascular system.

## Figures and Tables

**Table 1:** Quantitative analysis of **LDC** extracts by NMR spectroscopy.

	Molecule	Amount (µmol/g)					
		LDC1	LDC2	LDC3	LDC4	LDC5	LDC6
**Amino acids**	**Isoleucine**	-	1.28 ± 0.04	-	3.56 ± 0.11	-	7.85 ± 0.24
	**Valine**	-	1.92 ± 0.06	-	5.63 ± 0.17	-	11.40 ± 0.34
	**Alanine**	22.92 ± 0.69	11.60 ± 0.35	30.90 ± 0.93	26.37 ± 0.79	30.11 ± 0.90	23.62 ± 0.71
	**GABA**	59.77 ± 1.79	3.84 ± 0.12	134.24 ± 4.03	21.33 ± 0.64	-	-
	**Glutamate**	-	15.12 ± 0.45	-	-	-	-
	**Glutamine**	-	17.04 ± 0.51	-	-	-	-
	**Tyrosine**	-	-	-	1.67 ± 0.05	-	-
	**Phenylalanine**	-	1.68 ± 0.05	-	2.67 ± 0.08	-	10.53 ± 0.32
	**Tryptophan**	-	1.92 ± 0.06	-	5.78 ± 0.17	-	9.51 ± 0.29
**Organic acids**	**Lactic acid**	-	14.08 ± 0.42	42.51 ± 1.28	14.37 ± 0.43	-	-
	**Quinic acid**	104.92 ± 3.15	66.00 ± 1.98	869.41 ± 26.08	285.33 ± 8.56	-	-
	**Malic acid**	369.92 ± 11.10	93.60 ± 2.81	896.94 ± 26.91	430.00 ± 12.90	309.38 ± 9.28	202.64 ± 6.08
	**Citric acid**	-	16.68 ± 0.50	603.18 ± 18.10	103.33 ± 3.10	-	-
	**Succinic acid**	-	59.76 ± 1.79	857.24 ± 25.72	18.28 ± 0.55	-	51.11 ± 1.53
	**Fumaric acid**	8.54 ± 0.26	2.76 ± 0.08	-	-	-	-
	**Formic acid**	1.38 ± 0.04	1.20 ± 0.04	3.76 ± 0.11	3.11 ± 0.09	5.45 ± 0.16	1.36 ± 0.04
**Phenols**	**Gallic acid**	5.19 ± 0.16	3.72 ± 0.11	67.53 ± 2.03	2.56 ± 0.08	51.05 ± 1.53	91.81 ± 2.75
	**p-Coumaric acid**	-	5.16 ± 0.15	-	-	-	-
	**Caffeic acid**	-	7.20 ± 0.22	-	-	-	-
	**Catechin**	-	3.72 ± 0.11	18.12 ± 0.54	-	283.64 ± 8.51	171.40 ± 5.14
	**Glycosylated flavonoids (eq Q3G)**	20.69 ± 0.62	9.84 ± 0.30	90.51 ± 2.72	-	-	-
**Carbohydrates**	**Sucrose**	18.23 ± 0.55	-	-	-	-	190.42 ± 5.71
	**Glucose**	3497 ± 104	123.36 ± 3.70	613.65 ± 18.41	11081 ± 332	1468.15 ± 44.04	309.74 ± 9.29
	**Fructose**	-	36.00 ± 1.08	597.65 ± 17.93	-	242.84 ± 7.29	234.34 ± 7.03
**Miscellaneous**	**2,3 Butanediol**	-	2.72 ± 0.08	205.41 ± 6.16	-	-	17.74 ± 0.53
	**Nicotinamide**	0.46 ± 0.01	0.72 ± 0.02	7.29 ± 0.22	3.33 ± 0.10	5.24 ± 0.16	1.13 ± 0.03

**LDC1** (skin extract after rosé vinification process), **LDC2** (skin extract after red vinification process), **LDC3** (freeze-dried red wine), **LDC4** (freeze-dried red must), **LDC5** (seed extract after rosé vinification), **LDC6** (seed extract after red vinification process).

## Supplementary Materials

The following are available online at https://www.mdpi.com/1424-8247/13/5/87/s1. Table S1. ^1^H NMR resonance assignment of LDC samples; Figure S1: 1D ^1^H NMR spectrum of LDC1; Figure S2: ^1^H-^1^H TOCSY NMR spectrum of LDC1; Figure S3: 1D ^1^H NMR spectrum of LDC2; Figure S4: ^1^H-^1^H TOCSY NMR spectrum of LDC2; Figure S5: 1D ^1^H NMR spectrum of LDC4; Figure S6: 1D ^1^H NMR spectrum of LDC5; Figure S7: ^1^H-^1^H TOCSY NMR spectrum of LDC5; Figure S8: 1D ^1^H NMR spectrum of LDC6.
