# Supplementary material for: Vasorelaxant Effects Induced by Red Wine and Pomace Extracts of Magliocco Dolce cv
Source: Pharmaceuticals (Basel). 2020 May 3;13(5):87. doi: 10.3390/ph13050087 (PMC7281491; doi:10.3390/ph13050087)
Supplement: Supplementary file 1 [file pharmaceuticals-13-00087-s001.pdf]

Vasorelaxant Effects induced by Red Wine and Pomace extracts of Magliocco dolce *cv.*

Gabriele Carullo[1], Amer Ahmed[2], Fabio Fusi[3], Fabio Sciubba[4], Maria Enrica Di Cocco[4], Donatella Restuccia[1], Umile Gianfranco Spizzirri[1], Simona Saponara[2]* and Francesca Aiello[1]*

[1] Department of Pharmacy, Health and Nutritional Sciences, Department of Excellence 2018-2022, University of Calabria, Ed. Polifunzionale, 87036 Rende (CS), Italy; gabriele.carullo@unical.it (G.C.); donatella.restuccia@unical.it (D.R.); g.spizzirri@unical.it (U.G.S.); francesca.aiello@unical.it (F.A.).

[2] Department of Life Sciences, University of Siena, Via Aldo Moro 2, 53100 Siena, Italy; aa.biotechiub@gmail.com (A.A.); simona.saponara@unisi.it (S.S.).

[3] Department of Biotechnology, Chemistry and Pharmacy, Department of Excellence 2018-2022, University of Siena, Via Aldo Moro 2, 53100 Siena, Italy; fabio.fusi@unisi.it (F.F.).

[4] Department of Chemistry, University of Rome La Sapienza, Piazzale Aldo Moro 5, 00185 Roma, Italy; fabio.sciubba@uniroma1.it (F.S.); mariaenrica.dicocco@uniroma1.it (M.E.D.C.).

* Correspondence: simona.saponara@unisi.it (S.S.); francesca.aiello@unical.it (F.A.)

Table S1. ^{1}H NMR resonance assignment of LDC samples.

Compound	Assignment[a]	^{1}H (ppm)	Multiplicity[b]	Sample
**Amino acids**				
Valine	α -CH<br>β-CH<br>**γ-CH$_3$**<br>γ′-CH$_3$	3,62<br>2,29<br>**1,05**<br>1,00	m<br>m<br>**d**<br>d	2,4,6
Isoleucine	α -CH<br>β-CH<br>γ-CH<br>γ'-CH<br>**δ-CH$_3$**<br>δ′-CH$_3$	3,69<br>1,99<br>1,25<br>1,49<br>**1,01**<br>0,95	m<br>m<br>m<br>m<br>**d**<br>t	2,4,6
Alanine	α-CH<br>**β-CH$_3$**	3,77<br>**1,49**	q<br>**d**	1,2,3,4,5,6
GABA	α-CH$_2$<br>β-CH$_2$<br>**γ-CH$_2$**	2,35<br>1,91<br>**3,02**	t<br>m<br>**t**	1,2,3,4
Glutamate	α-CH<br>β-CH$_2$<br>γ-CH$_2$	α-CH<br>β-CH$_2$<br>**γ-CH$_2$**	3,74<br>2,08<br>**2,36**	2
Glutamine	α-CH<br>β-CH$_2$<br>γ-CH$_2$	α-CH<br>β-CH$_2$<br>**γ-CH$_2$**	3,76<br>2,11<br>**2,45**	2
Tyrosine	C2,6H-ring<br>**C3,5H-ring**	6,88<br>**7,18**	m<br>**m**	4
Phenylalanine	C2,6H-ring<br>CH-4 ring<br>**C3,5H-ring**	7,33<br>7,39<br>**7,42**	m<br>m<br>**m**	2,4,6
Tryptophan	CH-2 ring<br>CH-4 ring<br>CH-5 ring<br>**CH-6 ring**<br>CH-7 ring	7,31<br>7,27<br>7,19<br>**7,72**<br>7,53	s<br>d<br>m<br>**m**<br>d	2,4,6
Organic Acids				

Lactic acid	α-CH<br>**β-CH**	4,12<br>**1,37**	q<br>**d**	2,3,4
Quinic Acid	**CH_2-1**<br>CH-2<br>CH-3<br>CH-4<br>CH_2-5	**1,89, 2,09**<br>4,02<br>3,55<br>4,15<br>2,00, 2,06	**dd**<br>m<br>m<br>m<br>dd	1,2,3,4
Succinic acid	**α,β-CH_2**	**2,41**	**s**	2,3,4,6
Malic acid	**α –CH**<br>β-CH_2<br>β′-CH_2	**4,28**<br>2,34<br>2,65	**dd**<br>dd<br>dd	1,2,3,4
Fumaric acid	**α,β-CH=CH**	**6,51**	**s**	1,2
p-Coumaric acid	**α –CH**<br>β-CH<br>CH-2,6<br>CH-3,5	**6,36**<br>7,49<br>6,80<br>7,51	**d**<br>d<br>m<br>m	2
Gallic acid	**CH-2,6**	**7.05**	**s**	1,2,3,4,5,6
Caffeic acid	**α –CH**<br>β-CH<br>CH-2<br>CH-5<br>CH-6	**6,42**<br>7,42<br>7.22<br>6.96<br>7.14	**d**<br>d<br>d<br>d<br>dd	2
Formic acid	**CH**	**8,46**	**s**	1,2,3,4,5,6
Carbohydrates				
α-Glucose	**CH-1**<br>CH-2<br>CH-3<br>CH-4<br>CH-5<br>CH_2-6	**5,23**<br>3,55<br>3,72<br>3,42<br>3,84<br>3,73 – 3,90	**d**<br>m<br>m<br>m<br>m<br>m	1,2,3,4,5,6
β-Glucose	CH-1<br>**CH-2**<br>CH-3<br>CH-4<br>CH-5<br>CH_2-6	4,65<br>**3,24**<br>3,50<br>3,42<br>3,48<br>3,74 – 3,91	d<br>**dd**<br>m<br>m<br>m<br>m	1,2,3,4,5,6

Sucrose	**GLC CH-1**<br>CH-2<br>CH-3<br>CH-4<br>CH-5<br>CH2-6<br>FRU CH_2-1′<br>CH-3′<br>CH-4′<br>CH-5′<br>CH_2-6	**5,42**<br>3,59<br>3,79<br>3,48<br>3,85<br>3,82<br>3,69<br>4,22<br>4,06<br>3,90<br>3,82	**d**<br>m<br>m<br>m<br>m<br>m<br>m<br>d<br>m<br>m<br>m	1,6
Fructose	CH_2-1<br>**CH-3**<br>CH-4<br>CH-5<br>CH_2-6	3,69<br>**4,20**<br>4,06<br>3,90<br>3,82	m<br>**d**<br>m<br>m<br>m	2,3,5,6
Miscellaneous Compounds				
2,3-butanediol	**CH_3-1**<br>CH-2<br>CH-3<br>CH_3-4	**1,16**<br>3,65<br>3,74<br>1,17	**d**<br>m<br>m<br>d	2,3,6
Catechin	CH-6<br>**CH-8**<br>CH-2′<br>CH-5′<br>CH-6′<br>CH-2′′<br>CH-3′′<br>CH2-4′′	6,12<br>**6,14**<br>6,95<br>6,75<br>6,68<br>4,56<br>3,97<br>2,49, 2,82	d<br>**d**<br>d<br>d<br>dd<br>d<br>m<br>dd	2,3,5,6
Quercetin-3-O-Galactoside	CH-6<br>CH-8<br>CH-2′<br>CH-5′<br>CH-6′<br>**CH-1′′**<br>CH-2′′<br>CH-3′′<br>CH-4′′	6,20<br>6,41<br>7,69<br>6,91<br>7,55<br>**5,48**<br>3,80<br>3,87<br>3,98	d<br>d<br>d<br>d<br>dd<br>**d**<br>m<br>m<br>m	1,2,3

	CH-5′′<br>CH2-6′′	4,08<br>3,73	m<br>m	
Nicotinamide riboside	**C2H Nam**<br>C4H Nam<br>C5H Nam<br>C6H Nam	**9,61**<br>8,89<br>8,31<br>9,34	**s**<br>m<br>m<br>m	1,2,3,4,5,6

[a] specific resonance signal used for quantization are reported in bold

[b] s: singlet; d: doublet; dd: double doublet; t: triplet; q: quartet; m: multiplet

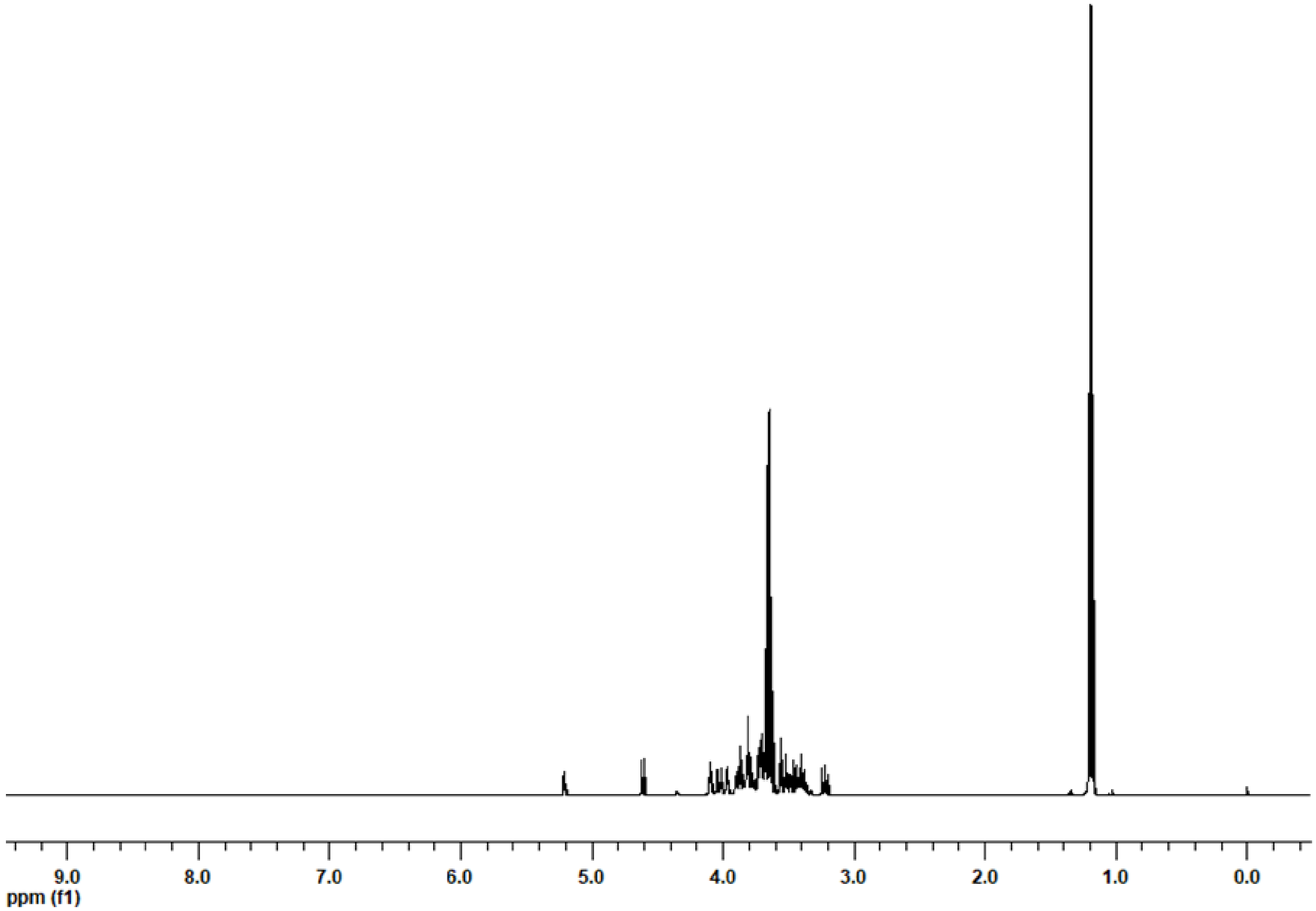

Figure S1: 1D ^{1}H NMR spectrum of LDC1

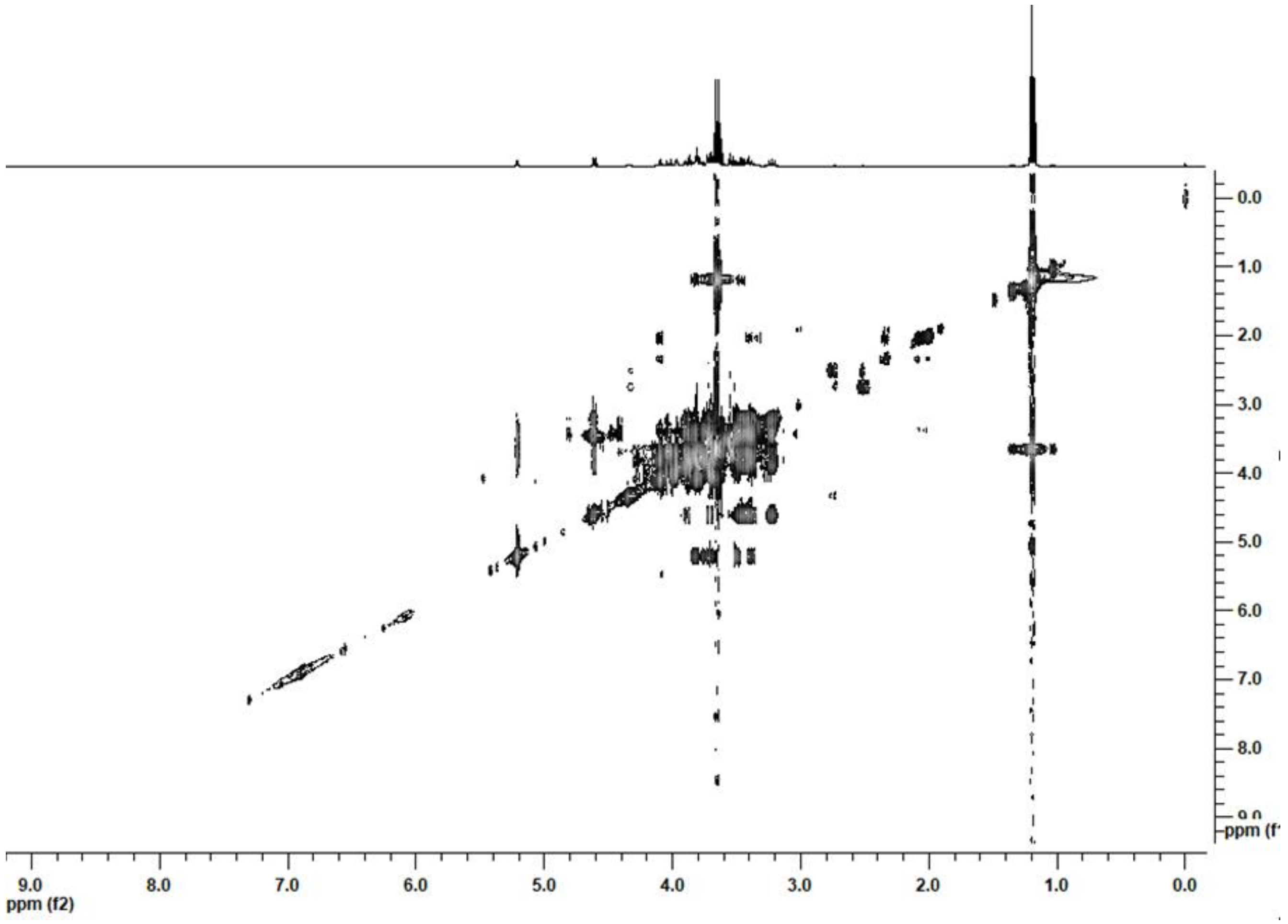

Figure S2: ^1H-^1H TOCSY NMR spectrum of LDC1

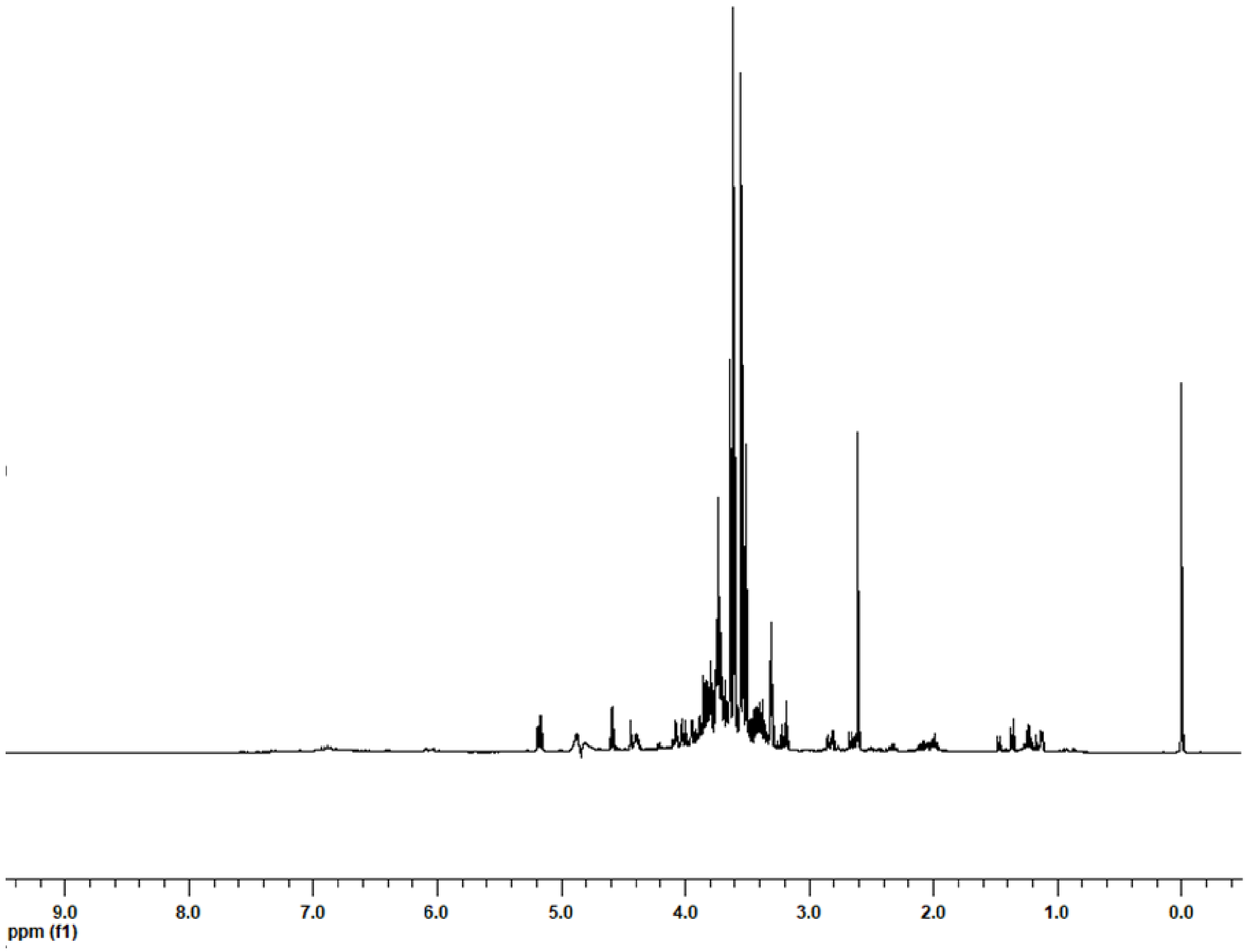

Figure S3: 1D ^{1}H NMR spectrum of LDC2

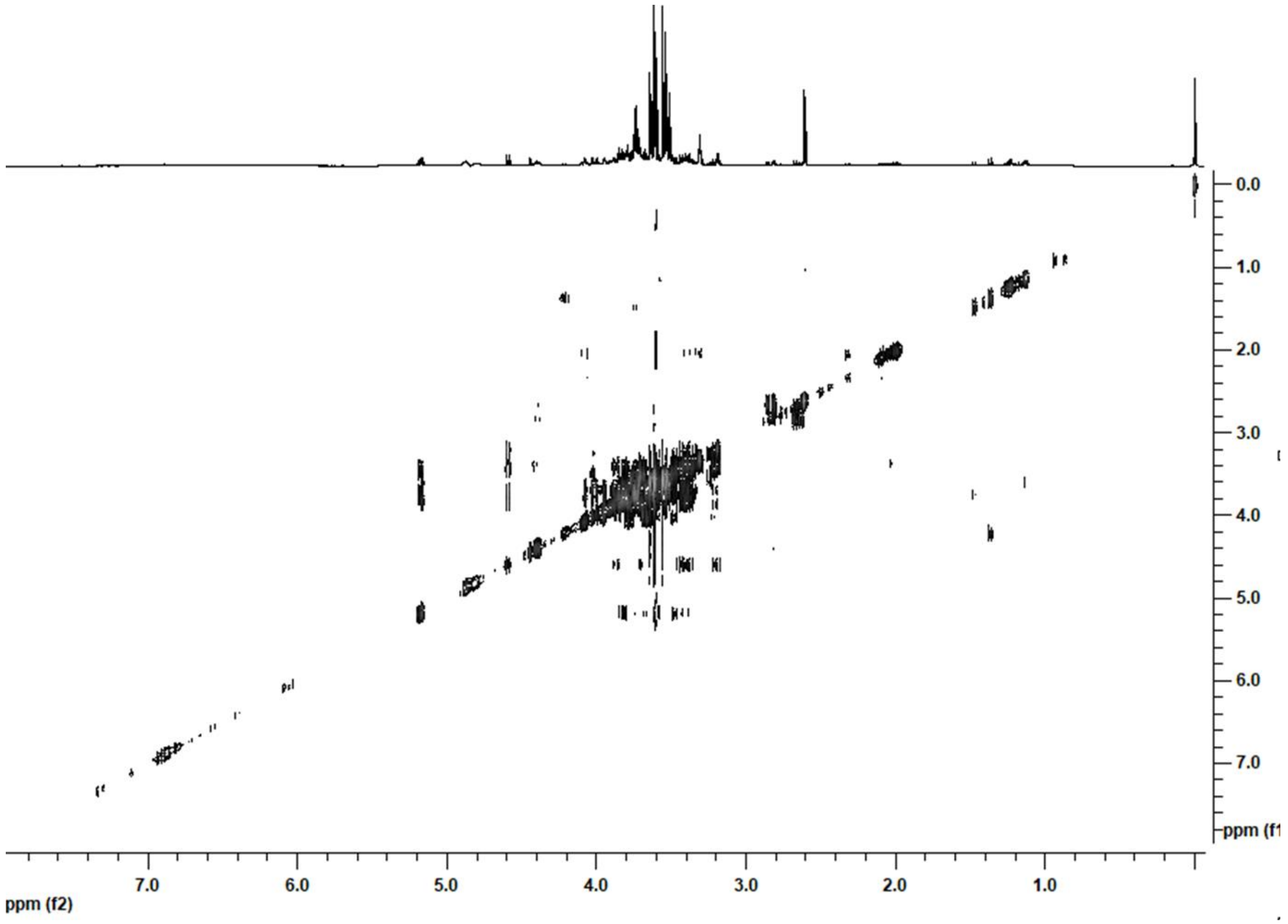

Figure S4: ^{1}H-^{1}H TOCSY NMR spectrum of LDC2

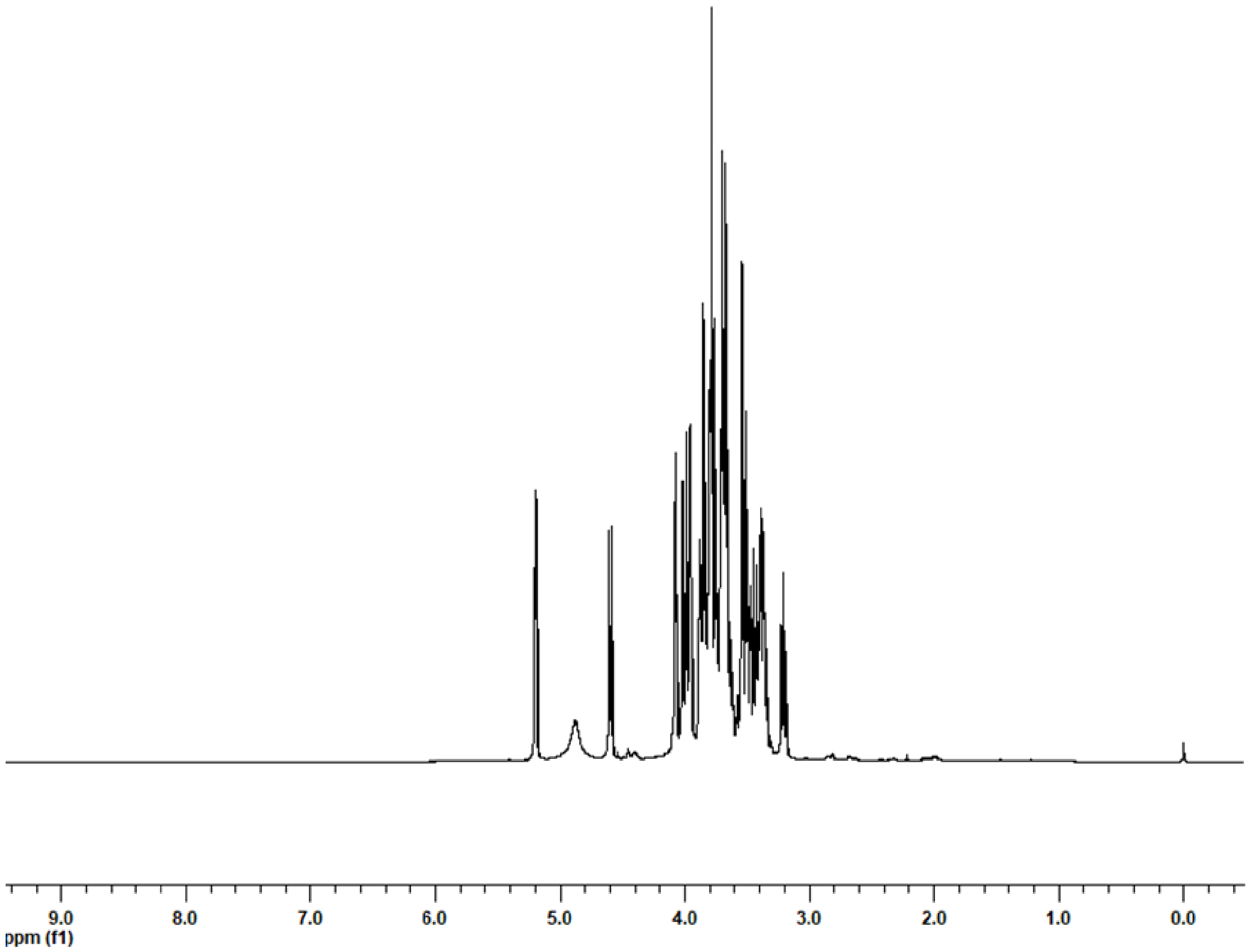

Figure S5: 1D ^{1}H NMR spectrum of LDC4

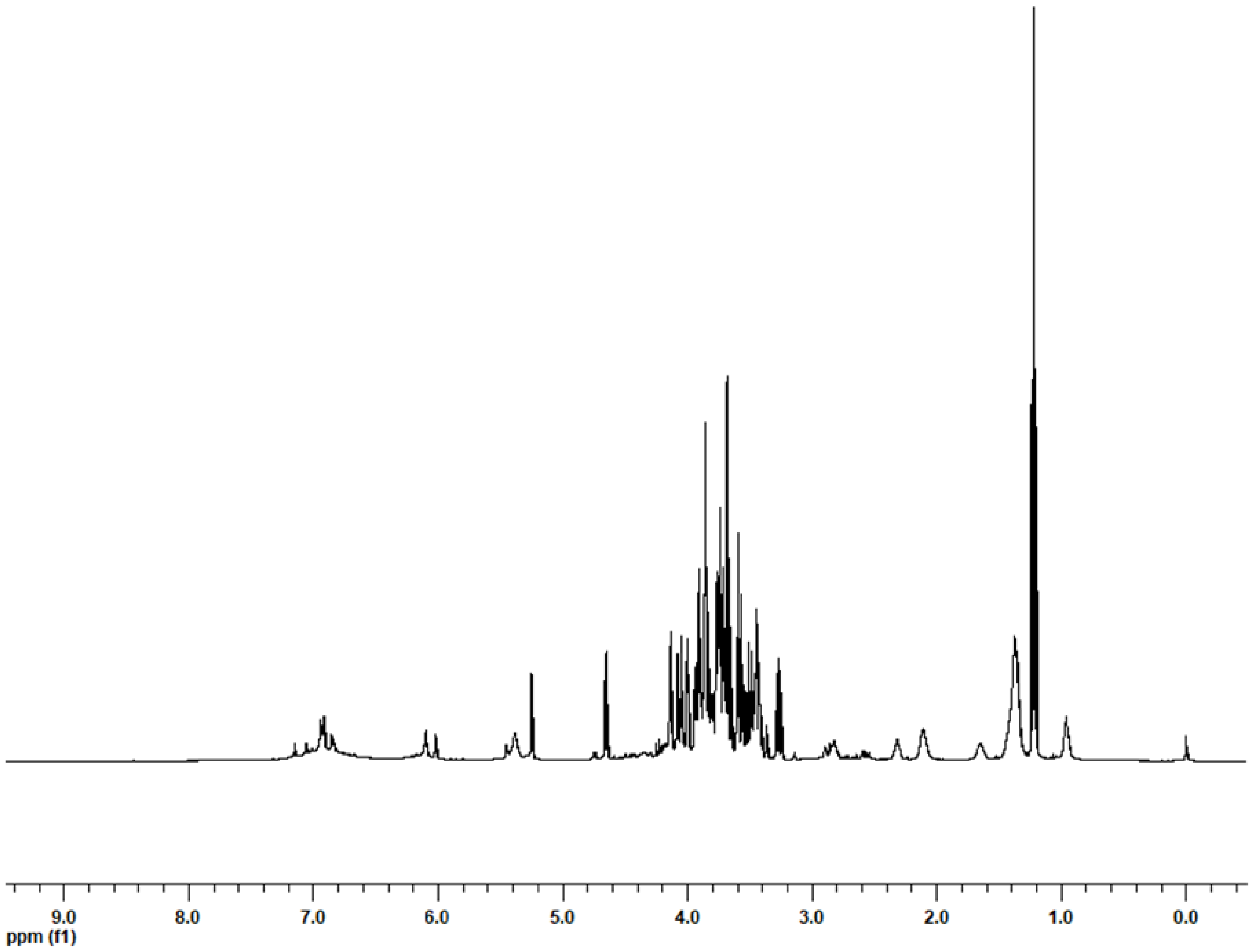

Figure S6: 1D ^{1}H NMR spectrum of LDC5

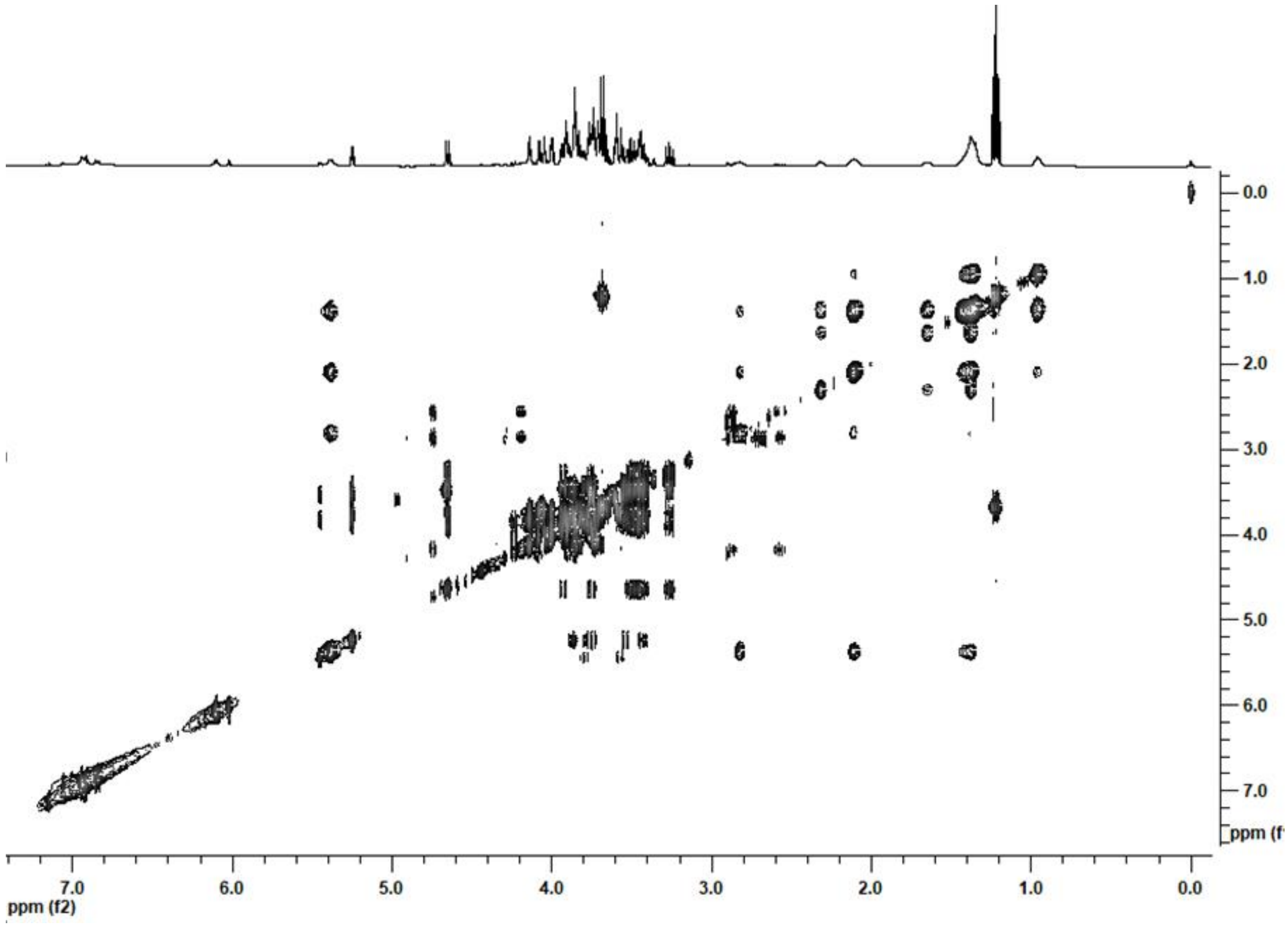

Figure S7: ^{1}H-^{1}H TOCSY NMR spectrum of LDC5

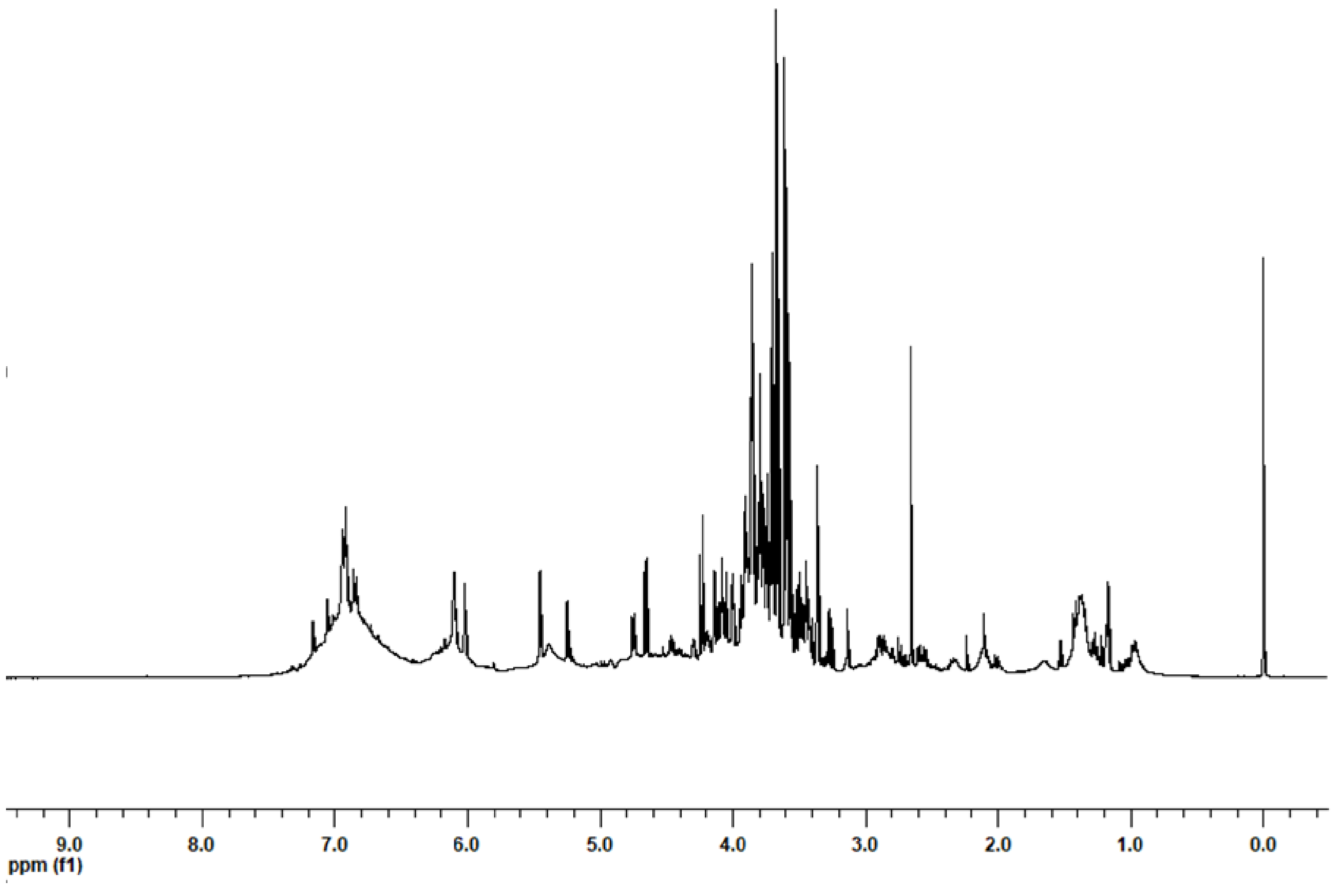

Figure S8: 1D ^{1}H NMR spectrum of LDC6
